# Different environmental gradients associated to the spatiotemporal and genetic pattern of the H5N8 highly pathogenic avian influenza outbreaks in poultry in Italy

**DOI:** 10.1111/tbed.13661

**Published:** 2020-07-02

**Authors:** Francesca Scolamacchia, Paolo Mulatti, Matteo Mazzucato, Marco Barbujani, William T. Harvey, Alice Fusaro, Isabella Monne, Stefano Marangon

**Affiliations:** ^1^ Istituto Zooprofilattico Sperimentale delle Venezie Legnaro (Padua) Italy; ^2^ Boyd Orr Centre for Population and Ecosystem Health Institute of Biodiversity, Animal Health and Comparative Medicine College of Medical, Veterinary and Life Sciences University of Glasgow Glasgow UK

**Keywords:** avian influenza, environmental drivers, epidemiology, H5N8 subtype, infectious disease outbreaks, multivariate statistics

## Abstract

Comprehensive understanding of the patterns and drivers of avian influenza outbreaks is pivotal to inform surveillance systems and heighten nations’ ability to quickly detect and respond to the emergence of novel viruses. Starting in early 2017, the Italian poultry sector has been involved in the massive H5N8 highly pathogenic avian influenza epidemic that spread in the majority of the European countries in 2016/2017. Eighty‐three outbreaks were recorded in north‐eastern Italy, where a densely populated poultry area stretches along the Lombardy, Emilia‐Romagna and Veneto regions. The confirmed cases, affecting both the rural and industrial sectors, depicted two distinct epidemic waves. We adopted a combination of multivariate statistics techniques and multi‐model regression selection and inference, to investigate how environmental factors relate to the pattern of outbreaks diversity with respect to their spatiotemporal and genetic diversity. Results showed that a combination of eco‐climatic and host density predictors were associated with the outbreaks pattern, and variation along gradients was noticeable among genetically and geographically distinct groups of avian influenza cases. These regional contrasts may be indicative of a different mechanism driving the introduction and spreading routes of the influenza virus in the domestic poultry population. This methodological approach may be extended to different spatiotemporal scale to foster site‐specific, ecologically informed risk mitigating strategies.

## 1 INTRODUCTION

In 2017, the Italian poultry sector has been involved in the highly pathogenic avian influenza (HPAI) epidemic caused by type A (H5N8) viruses of clade 2.3.4.4 that spread across 29 European countries from the winter of 2016 (Brown et al., 2017). In Italy, eighty‐three outbreaks were confirmed in the domestic poultry during 2017, that peculiarly occurred in two distinct epidemic waves, affecting both the rural and the industrial sectors (Mulatti et al., 2018). The first wave, lasting between January and May 2017, comprised 16 cases in holdings located on the fringe of the densely populated poultry area (DPPA) in northeast Italy. The large majority of affected premises (*n* = 14, 87.5%) were close to wetlands, where substantial populations of wild waterfowl were reported. Contact tracing reports did not identify at‐risk contacts between infected farms, and sequenced viruses revealed considerable nucleotide differences (Fusaro et al., 2017; Mulatti et al., 2018). The second epidemic wave started in July, 50 days after the last reported case. Sixty‐seven farms in the DPPA located in the Po Valley were affected, with sparse incursions into western and central Italy. Up to late September, epidemiological and genetic characteristics of the cases were comparable to those of the outbreaks detected in the first wave. Contrarily, since October, assessment of the contact network highlighted that multiple contact types (e.g. neighbourhood risks, indirect contacts via contaminated vehicles, movements of feed lorries, contacts via shared personnel) occurred between infected farms. Moreover, phylogenetic analyses revealed 99%–100% similarity between viruses isolated in those premises. Only a smaller fraction of outbreaks were reported occurring in proximity to wetlands (*n* = 39, 58.21%) compared to the first wave. Notably, wild bird populations appeared to be only marginally involved during the entire epidemic, with only 12 H5N8 HPAI cases confirmed.

The transmission pattern during that epidemic was likely driven by distinct dynamic interactions between hosts and the environment where avian influenza (AI) viruses circulated, and these interactions shaped the unique epidemiological characteristics of the first and second wave. Hence, in order to possibly improve control strategies, a better understanding of how ecological interactions among poultry populations, viruses and their environment can be related to the pattern of disease emergence is needed. In nature, distribution of (bio)diversity is highly non‐random and the pattern that will result is often interpretable in terms of environmental gradients, which impose some structure to the ecological communities (Worm & Tittensor, 2018). Empirically measured environmental conditions can be related to both the driving factors that may cause a pattern to exist, and to the key mechanism(s) that limits or promotes diversity in coexisting populations of an ecological community. The complex systems studied in ecology and epidemiology are often remarkably similar, with respect to the multifactorial interactions with the environment as a whole that shape the distribution, structure, abundance and demography of animals and plants communities as well as disease emergence and persistence. Therefore, studying the relationships between environmental factors and the pattern of infectious disease events can provide clues to the mechanism(s) underlying the pathogen introduction route(s) into the host community.

Avian influenza circulates in two main systems. The first is the wild bird ecosystem, where all influenza virus subtypes are maintained in the low pathogenic forms, characterized by a predominantly environmental transmission between hosts, especially within residential and migrating populations of the orders *Anseriformes* and *Charadriiformes*, through sharing foraging and breeding areas. The second is the poultry farms and their associated value‐chain networks system, which forms a secondary system. In the latter, once the AI virus is introduced, its transmission is mainly related to human‐mediated activities (Alexander, 2007). Environmental correlates of avian influenza virus occurrence and persistence have been broadly studied at local and global scale, both in wild birds and domestic poultry populations, by means of spatiotemporal and niche suitability modelling, especially for the H5N1 HPAI virus, which has spread extensively and for more than a decade worldwide (FAO, 2019). Several spatial analytical studies were reviewed by Gilbert and Pfeiffer (Gilbert & Pfeiffer, 2012 and references therein). These authors highlighted three main categories of factors, whose pattern associated with H5N1 HPAI presence showed to be consistent worldwide: (a) high poultry density, with a specific indication of the domestic waterfowls productive type; (b) anthropogenic variables (human population density, distance to roads and cities, which can be a proxy for indirect contacts due to human‐mediated activities, movements of poultry, poultry products, personnel, manure, equipment); and (c) presence of water or indices of habitat suitable for waterfowl. Besides, AI occurrence has been found associated to a wide range of values of eco‐climatic variables (e.g. vegetation indices, temperature, precipitation) aggregated over different periods (month, year, season), reflecting the plasticity of this virus in nature and supporting the hypothesis that, for a directly transmitted disease, such factors did not represent a constraint. Land cover and land use were rarely systematically included and, if so, they define a combination of previously described factors (environment suitable for wild birds’ ecology or human related activities) (Gilbert & Pfeiffer, 2012). Finally, low altitude values were found as well consistently associated to HPAI outbreaks occurrence (Loth et al., 2011; Mannelli, Ferre, & Marangon, 2006). Conversely, for the H5Nx viruses of clade 2.3.4.4 there is a notable paucity of such studies and most of them have a focus on local production systems (Dhingra et al., 2016; Guinat et al., 2019; Guinat et al., 2018; Kim et al., 2018), largely because at the time of publication, extensive data on these viruses potential range were not yet available. The most noticeable difference concerned the prevalent effect of host distribution, especially of extensively reared poultry, on H5N1 HPAI outbreaks occurrence, versus the association of H5Nx clade 2.3.4.4 viruses with a mixture of environmental, managerial (biosecurity practices) and host distribution variables, especially chicken intensively reared.

It is hypothesized that in domestic poultry populations, the risk of introduction is determined by the farm's neighbourhood characteristics, contact network and the levels of biosecurity. Therefore, if wild birds act as the main source of infection for domestic poultry, then the disease pattern is expected to be strongly associated to eco‐climatic and physical environmental factors that directly or indirectly promote the availability of shelter and food for waterfowl. Contrarily, any anthropogenic, managerial and demographic aspects or their surrogate measures that are found associated to outbreaks occurrence or outbreaks’ features could be deemed as a robust indication that a secondary transmission between domestic premises is at the origin of the infection.

Recently, it was postulated that assembling methodologies from diverse disciplines (ecology, epidemiology and phylogenetics) can advance the understanding of emerging infectious diseases, which are inherently difficult to study because of ecological interactions among animal populations, viruses and their environment (Plowright, Sokolow, Gorman, Daszak, & Foley, 2008). The combined application of statistical techniques from multivariate data analysis (ordination and clustering methods) and classical regression modelling, proved well suited to serve that aim for avian influenza (Carrel, Emch, Nguyen, Todd Jobe, & Wan, 2012; Mughini‐Gras et al., 2014). Data reduction is the first step in a complete analysis of ecological information, usually preceding the possibility to examine the ordination‐environment relationship(s). Ordination is a well‐established strategy for reducing the dimensionality of a multivariate data set by condensing a large number of original variables into a smaller set of new dimensions with a minimum loss of information. It has long been used in ecology for both data presentation and to reveal important and interpretable environmental gradients associated with the community data (Legendre & Legendre, 2012). By means of ordination, ecological community attributes can be summarized and plotted in a multidimensional graph, which can help us see whether the community data are structured or contain patterns. These patterns may reflect a community's response to multiple environmental changes or more subtle biological interactions. Besides, with the advent of phylogeography, integrating the genetic evolution dimension in the study of the ecological system within which AI viruses occur and evolve became easier.

Our goal in the present study was twofold. Firstly, to determine whether it was possible to identify a pattern in the Italian H5N8 HPAI outbreaks diversity, with respect to their peculiar spatiotemporal and genetic features, using multivariate statistical methods. Secondly, to reveal whether the pattern of the outbreaks community could be related to eco‐environmental variables gradients, through multi‐model regression selection and inference. Once the pattern of H5N8 HPAI outbreaks in Italy will be related to environmental factors variation, this may prove useful in developing site‐specific risk mitigating measures.

## 2 MATERIALS & METHODS

### 2.1 Outbreaks data

The data set used to explore the environmental correlates of AI outbreaks pattern consists of the 83 H5N8 HPAI cases confirmed in the domestic poultry in Italy in 2017. Table 1 summarizes the main features of each outbreak as derived from the epidemiological investigation and genetic characterization of the virus isolates, which have been thoroughly described by Mulatti et al., (2018) and by Fusaro et al., (2017).

**Table 1:** Information on the 83 H5N8 HPAI outbreaks occurred in domestic poultry during the 2017 epidemic in Italy

Outbreak ID	Cluster^1^	Region	Province	Poultry production sector^2^	Common name of poultry species/production type	Symptoms onset	Virus type^3^	Epidemic wave^4^	Outbreak type^5^
1		Veneto	VENEZIA	Industrial	Turkey/meat	17/01/2017	India	1	1
2		Veneto	PADOVA	Industrial	Turkey/meat	23/01/2017	Poland	1	1
3		Veneto	ROVIGO	Industrial	Chicken/egg	23/01/2017	Poland	1	1
4		Emilia‐Romagna	PARMA	Industrial	Turkey/meat	01/02/2017	Croatia	1	1
5		Lombardy	MANTOVA	Industrial	Turkey/meat	13/02/2017	Croatia	1	1
6		Veneto	VERONA	Industrial	Turkey/meat	16/02/2017	Poland	1	1
7		Lombardy	MANTOVA	Industrial	Turkey/meat	21/02/2017	Croatia	1	1
8		Veneto	VENEZIA	Rural	Multispecies/mixed	27/02/2017	Croatia	1	1
9		Veneto	VENEZIA	Rural	Multispecies/mixed	27/02/2017	Croatia	1	1
10		Veneto	TREVISO	Rural	Multispecies/mixed	16/03/2017	Poland	1	1
11		Veneto	VERONA	Industrial	Turkey/meat	27/03/2017	Poland	1	1
12		Friuli‐Venezia Giulia	PORDENONE	Rural	Multispecies/mixed	28/03/2017	Poland	1	1
13		Piedmont	TORINO	Rural	Multispecies/mixed	20/03/2017	Poland	1	1
14		Emilia‐Romagna	BOLOGNA	Industrial	Chicken/egg	05/04/2017	Poland	1	1
15		Veneto	VERONA	Industrial	Turkey/meat	09/04/2017	Poland	1	1
16		Lombardy	MANTOVA	Industrial	Turkey/meat	27/05/2017	Italy A	1	1
17		Lombardy	MANTOVA	Industrial	Turkey/meat	17/07/2017	Italy A	2	1
18		Lombardy	MANTOVA	Rural	Mallard duck/mixed	17/07/2017	Italy A	2	1
19	Man	Lombardy	MANTOVA	Industrial	Chicken/egg	19/07/2017	Italy A	2	1
20		Veneto	VERONA	Industrial	Turkey/meat	23/07/2017	Italy A	2	1
21		Veneto	VERONA	Industrial	Turkey/meat	23/07/2017	Italy A	2	1
22		Lombardy	MANTOVA	Industrial	Chicken/egg	27/07/2017	Italy A	2	1
23		Veneto	VERONA	Industrial	Turkey/meat	28/07/2017	Italy A	2	1
24		Emilia‐Romagna	PARMA	Industrial	Turkey/meat	31/07/2017	Italy A	2	1
25	Man	Lombardy	MANTOVA	Industrial	Turkey/meat	04/08/2017	Italy A	2	2
26	Man	Lombardy	MANTOVA	Industrial	Turkey/meat	29/07/2017	Italy A	2	2
27	Man	Lombardy	MANTOVA	Industrial	Turkey/meat	28/07/2017	Italy A	2	2
28		Lombardy	PAVIA	Industrial	Goose/meat	03/08/2017	Italy B	2	1
29	Man	Lombardy	MANTOVA	Industrial	Turkey/meat	03/08/2017	Italy A	2	2
30		Veneto	VERONA	Industrial	Turkey/meat	19/08/2017	Italy A	2	1
31		Lombardy	LODI	Rural	Multispecies/mixed	10/08/2017	Italy B	2	1
32		Veneto	VERONA	Industrial	Turkey/meat	20/08/2017	Italy A	2	1
33		Veneto	VERONA	Industrial	Turkey/meat	20/08/2017	Italy A	2	1
34		Lombardy	PAVIA	Rural	Chicken/mixed	16/08/2017	Italy B	2	1
35		Veneto	VERONA	Industrial	Turkey/meat	27/08/2017	Italy A	2	1
36		Lombardy	CREMONA	Industrial	Turkey/meat	01/09/2017	Italy B	2	1
37		Veneto	PADOVA	Rural	Multispecies/mixed	08/09/2017	Italy A	2	1
38		Veneto	VICENZA	Industrial	Turkey/meat	20/09/2017	Italy A	2	1
39		Veneto	VERONA	Industrial	Turkey/meat	25/09/2017	Italy A	2	1
40	Vic	Veneto	VICENZA	Industrial	Multispecies/mixed	24/09/2017	Italy A	2	1
41		Lombardy	CREMONA	Rural	Multispecies/mixed	15/09/2017	Italy B	2	1
42		Emilia‐Romagna	FERRARA	Industrial	Chicken/egg	04/10/2017	Italy B	2	1
43	Vic	Veneto	VICENZA	Industrial	Chicken/meat	02/10/2017	Italy A	2	2
44	Ber	Lombardy	BERGAMO	Rural	Chicken/egg	09/10/2017	Italy B	2	2
45	Vic	Veneto	VICENZA	Rural	Multispecies/meat	09/10/2017	Italy A	2	2
46	Ber	Lombardy	BERGAMO	Rural	Multispecies/mixed	11/10/2017	Italy B	2	1
47		Veneto	PADOVA	Industrial	Turkey/meat	11/10/2017	Italy A	2	1
48	Bre	Lombardy	BRESCIA	Industrial	Turkey/meat	09/10/2017	Italy B	2	1
49	Ber	Lombardy	BERGAMO	Rural	Multispecies/mixed	12/10/2017	Italy B	2	2
50	Bre	Lombardy	BRESCIA	Industrial	Turkey/meat	13/10/2017	Italy B	2	2
51		Lombardy	SONDRIO	Rural	Chicken/meat	12/10/2017	Italy B	2	2
52		Lombardy	MANTOVA	Industrial	Turkey/meat	12/10/2017	Italy A	2	1
53		Veneto	PADOVA	Rural	Multispecies/mixed	13/10/2017	Italy A	2	1
54		Veneto	PADOVA	Rural	Multispecies/mixed	13/10/2017	Italy A	2	1
55	Bre	Lombardy	BRESCIA	Industrial	Turkey/meat	18/10/2017	Italy B	2	2
56	Bre	Lombardy	BRESCIA	Industrial	Turkey/meat	18/10/2017	Italy B	2	2
57	Ber	Lombardy	BERGAMO	Rural	Multispecies/mixed	20/10/2017	Italy B	2	2
58	Ber	Lombardy	MILANO	Rural	Chicken/egg	24/10/2017	Italy B	2	1
59	Bre	Lombardy	BRESCIA	Industrial	Mallard duck/meat	25/10/2017	Italy B	2	2
60	Bre	Lombardy	BRESCIA	Industrial	Chicken/meat	26/10/2017	Italy B	2	2
61	Bre	Lombardy	BRESCIA	Industrial	Chicken/meat	27/10/2017	Italy B	2	2
62	Bre	Lombardy	BRESCIA	Industrial	Turkey/meat	30/10/2017	Italy B	2	2
63	Bre	Lombardy	BRESCIA	Industrial	Turkey/meat	31/10/2017	Italy B	2	2
64		Lombardy	BERGAMO	Industrial	Turkey/meat	31/10/2017	Italy B	2	2
65	Bre	Lombardy	BRESCIA	Industrial	Turkey/meat	01/11/2017	Italy B	2	2
66	Bre	Lombardy	BRESCIA	Industrial	Turkey/meat	01/11/2017	Italy B	2	2
67	Bre	Lombardy	BRESCIA	Industrial	Mallard duck/meat	26/10/2017	Italy B	2	2
68	Bre	Lombardy	BRESCIA	Industrial	Mallard duck/meat	01/11/2017	Italy B	2	2
69	Bre	Lombardy	BRESCIA	Industrial	Turkey/meat	02/11/2017	Italy B	2	2
70	Bre	Lombardy	BRESCIA	Industrial	Chicken/egg	03/11/2017	Italy B	2	2
71		Piedmont	ASTI	Industrial	Chicken/egg	26/10/2017	Italy B	2	1
72	Bre	Lombardy	BRESCIA	Industrial	Chicken/breeder	07/11/2017	Italy B	2	2
73		Lazio	ROMA	Rural	Chicken/egg	02/11/2017	Italy B	2	1
74	Bre	Lombardy	CREMONA	Industrial	Turkey/meat	08/11/2017	Italy B	2	2
75	Bre	Lombardy	BRESCIA	Industrial	Chicken/egg	10/11/2017	Italy B	2	2
76	Bre	Lombardy	BRESCIA	Industrial	Chicken/egg	09/11/2017	Italy B	2	2
77	Bre	Lombardy	BRESCIA	Industrial	Chicken/egg	09/11/2017	Italy B	2	2
78	Bre	Lombardy	BRESCIA	Industrial	Chicken/meat	10/11/2017	Italy B	2	2
79	Bre	Lombardy	BRESCIA	Industrial	Chicken/egg	10/11/2017	Italy B	2	2
80	Bre	Lombardy	BRESCIA	Industrial	Chicken/meat	21/11/2017	Italy B	2	2
81		Veneto	TREVISO	Rural	Multispecies/mixed	07/11/2017	Italy A	2	1
82		Veneto	TREVISO	Industrial	Multispecies/mixed	30/11/2017	Italy A	2	1
83		Emilia‐Romagna	RAVENNA	Industrial	Turkey/meat	09/12/2017	Italy B	2	1

^1^ Primary (*n* = 4) and secondary (*n* = 32) infected premises that can be grouped into 4 different clusters inferable due to high number of connections (proximity between cases, genetic similarity and/or trade of living poultry (Mulatti et al., 2018)). Ber, Bergamo; Bre, Brescia; Man, Mantova; Vic, Vicenza

^2^ The industrial sector comprises holdings where birds are kept for commercial purposes only. The rural sector includes those holdings where birds (less than 250 in number) are reared and kept exclusively for self‐consumption or are reared and traded with other rural premises, or a combination of these activities (Cecchinato et al., 2011)

^3^ One of the four distinct genomic groups identified during the H5N8 HPAI epidemic in Italy (Fusaro et al., 2017; Mulatti et al., 2018)

^4^ First wave (1): January–May 2017; second wave (2): July–December 2017

^5^ As defined by (Mulatti et al., 2018), a *primary case* (1) is when no at‐risk contacts with previously detected outbreak emerged from the epidemiological investigation and no phylogenetic similarity was found. Contrarily, a *secondary case* (2) is when at‐risk contacts with an infected farm during its outbound risk period could be reliably traced back and/or genomic analyses showed a high degree of similarity between virus isolates.

### 2.2 Environmental data

By reviewing relevant literature, in particular for viruses of the H7 and H5 subtypes (such as H5N1 and H5NX clade 2.3.4.4), we identified factors, as well as proxies for some of these, known to define area suitability for HPAI outbreaks (Belkhiria, Alkhamis, & Martinez‐Lopez, 2016; Dhingra et al., 2016; Guinat et al., 2019), or that best relate to virus occurrence or virus introduction and spread in domestic poultry and wild birds, both at local or global‐scales (Gilbert & Pfeiffer, 2012; Si, de Boer, & Gong, 2013; Si et al., 2010). This information was then combined with an assessment of data availability, so that seventeen eco‐environmental factors were selected as inputs for our data analysis. Those variables were grouped into three main categories: host, land cover and eco‐climatic variables. For each factor/group of factors, corresponding data sources, trends relative to AI virus spread or risk of introduction, rationale for model inclusion, and references are described below and summarized in Table 2.

**Table 2:** List of the seventeen eco‐environmental predictors included in the regression model

Category	Description of variables	Measure computed	Source
Eco‐climatic variables	Vegetation indices		
	*EVI*	2 weeks mean value	MODIS
	*NDVI*	2 weeks mean value	MODIS
	Elevation	m above sea level	DTM
	Precipitation		
	Cumulative precipitation amount 15 days prior to onset of symptoms	total mm	Global Precipitation Measurement mission
	Number of days with ≥ 1mm rain in 15 days prior to onset of symptoms	number of days	Elaboration on GPM data
	Geodesic distance to the nearest wetland	m to nearest wetland	GIS data manipulation
Land cover variables	Land cover class (level 1)		
	Artificial surfaces	Per cent	Corine Land Cover 2012*, 2
	Agricultural areas	Per cent	Corine Land Cover 2012*, 2
	Rice fields (level 3)	Per cent	Corine Land Cover 2012*, 2
	Forest and semi‐natural areas	Per cent	Corine Land Cover 2012*, 2
	Wetlands	Per cent	Corine Land Cover 2012*, 2
	Water bodies	Per cent	Corine Land Cover 2012*, 2
Host variables	Poultry population density 3 km buffer zone	heads/km^2^	National Animal Registry (BDN); census data during outbreak investigation
	Poultry population density 10 km buffer zone	heads/km^2^	National Animal Registry (BDN); census data during outbreak investigation
	Poultry farm density 3 km buffer zone	farms/km^2^	National Animal Registry (BDN); census data during outbreak investigation
	Poultry farm density 10 km buffer zone	farms/km^2^	National Animal Registry (BDN); census data during outbreak investigation
	Human population density	people/km^2^	Italian National Institute of Statistics (ISTAT)^†^, ^1^

^†^ Data processed by Urbistat S.r.l (http://ugeo.urbistat.com/AdminStat/en/it/demografia/dati‐sintesi/italy/380/1).

* Corine Land Cover 2012 v18 (version 18 was updated in 2016).

Host variables include the densities of human population, of domestic poultry holdings and of their respective poultry population. Poultry farms and poultry population densities were considered a surrogate for the risk of virus spread from farm to farm associated to densely populated poultry areas, where the high direct and indirect contact rates in an immunologically naïve hosts’ community influence disease flare‐ups with known major detrimental effects (Marangon, Capua, Pozza, & Santucci, 2004). We used the official poultry census data, which were derived in 2017 from the National Animal Registry, coupled with actual census data, collected during each outbreak investigation. Densities were computed for each outbreak for a buffer radius of 3 and 10 km, in relation to the protection and surveillance zones put in place during an outbreak. The number of holdings that fell within the buffer zones and were in operation up to two weeks preceding the date of onset of symptoms (considered as the maximum incubation period for AI in a flock (Swayne, 2016), as well as their maximum stocking capacity, were included in the calculation. Human population density was included as a proxy for any human‐mediated transmission mechanisms, and was based on the data provided by the Italian National Institute of Statistics (ISTAT) at the level of municipal census block for the year 2017.

Land cover and eco‐climatic variables were extracted from a system developed at the Istituto Zooprofilattico Sperimentale delle Venezie (Legnaro, Italy; https://www.izsvenezie.com/) named *Environmental data for Veterinary Epidemiology* (EVE), which allows managing data from different sources, both in raster and table format. Different agro‐ecological conditions summarized by land cover and land use or cropping intensity areas have been found associated to the presence of AI or the risk of avian influenza spread (Hogerwerf et al., 2010; Iglesias, Muñoz, Martínez, & Torre, 2010; Loth et al., 2011). By offering a cardinal proxy for locally expected biodiversity and ecological processes, land cover mimics, with other habitat determinants (such as climate), the interface between human activities and natural environments. For the land cover data, we calculated the percentage of the classes included in the Corine Land Cover (CLC) 2012 data set (https://land.copernicus.eu/pan‐european/corine‐land‐cover/clc‐2012 ), which are grouped in a three‐level hierarchy (https://land.copernicus.eu/user‐corner/technical‐library/corine‐land‐cover‐nomenclature‐guidelines/html), and we assigned those values to the buffer area of 1 km around each outbreak. In our analyses, we used the five first‐level categories ‘artificial surfaces’, ‘agricultural areas’, ‘forest and semi‐natural areas’, ‘wetlands’ and ‘water bodies’ in order to capture coarse environmental gradients associated with the Italian HPAI H5N8 outbreaks. In addition to those classes, we considered the third‐level class category ‘rice fields’ (in the lowest hierarchical level of ‘agricultural areas’), as this, in the same way as permanent water bodies or surface water, was consistently found correlated to AI virus occurrences, because it implies the availability of natural resources for wild waterfowl (Gilbert & Pfeiffer, 2012; Si et al., 2010).

Eco‐climatic variables comprise two vegetation indices (Normalized Difference Vegetation Index—NDVI and Enhanced Vegetation Index—EVI), precipitation, elevation and distance to the nearest wetlands. Vegetation indices, although with inconsistent values across countries and studies, are necessary markers of the environment in which AI viruses may spread, especially in combination with other environmental factors such as altitude and precipitation anomalies (Gilbert & Pfeiffer, 2012). NDVI and EVI mean values for the two weeks preceding the onset of symptoms were computed for a buffer of 1 km around each outbreak location. In EVE, vegetation index data sets across Italian territory are modelled from the Moderate Resolution Imaging Spectroradiometer (MODIS) satellite‐derived data (https://lpdaac.usgs.gov/products/mod13a2v006/). Meteorological data, such as rainfall, were suggested to influence viral prevalence or persistence as well as wild birds behaviour (e.g. breeding ecology) (Gilbert & Pfeiffer, 2012 and references therein). Two measures concerning precipitation were considered: cumulative rainfall (totals mm) over two weeks preceding the onset of symptoms within 1 km buffer around each outbreak location, and the number of rainy days (i.e. a rainy day is considered any day with ≥1 mm rain as measured in 24 hr by a rainy gauge) in the same two weeks. For Italy, EVE’s precipitation data were derived from the daily GPM v.6 data set (https://www.nasa.gov/mission_pages/GPM/main/index.html). The average elevation of outbreak location (in a 1 km buffer area) was included, as it governs various types of vegetative growth, as does the degree and direction of slope, and can be considered an indicator of land cover. High‐elevation areas are usually dominated by forests‐like and permanent vegetation compared to low‐elevation areas, where grasses and herbaceous plants grow, where wetlands, rivers and canals lie, and where there is a mixture of intensive uses of the lands. Elevation data were acquired from the 20‐m resolution Digital Terrain Model from the Italian National Geoportal (http://www.pcn.minambiente.it/mattm/en/). Lastly, geodesic distance of each outbreak location to the nearest wetlands (open water such as sea or inland water points such as rivers, lakes, wetlands or canals) was used as a measure for water proximity to quantify potential direct or indirect contacts with wild birds. Values were derived from GIS data manipulation (QGIS Development Team, 2009, version 2.18).

### 2.3 Phylogenetic analysis

Pairwise evolutionary distances between domestic cases were calculated from the branch lengths of phylogenetic trees estimated using an alignment comprising one virus sequence associated with a bird belonging to each case. Aligned haemagglutinin (HA) gene sequences and dates of onset of symptoms were used to reconstruct time‐measured phylogenetic trees inferred using the program BEAST v1.10.4 (Suchard et al., 2018). Prior to implementation in BEAST, models of nucleotide evolution were compared using BIC and AIC calculated using jModelTest v2.1.7 (Darriba, Taboada, & Doallo, 2012). This indicated that sequence evolution should be modelled using the general time reversible model with a proportion of invariant sites and a gamma distribution describing among‐site rate variation with four categories estimated from the data (GTR + I + Γ_4_). This model was implemented in BEAST with a relaxed molecular clock with branch rates drawn from a lognormal distribution (Drummond, Ho, Phillips, & Rambaut, 2006). This model, where branch‐specific rates of evolution are drawn from a lognormal distribution, was favoured over a strict molecular clock and a relaxed model with branch rates drawn from an exponential distribution through comparison of AICM. A flexible Bayesian skyride demographic model with time‐aware smoothing (Minin VN & Bloomquist EW, 2008) was chosen over simpler coalescent models that make greater assumptions of population size through time (e.g. constant size, exponential growth). Phylogenies were constructed using an alignment of 83 sequences from each domestic case and a further ten sequences from wild birds cases and, alternatively, using an alignment of domestic cases only. For each alignment, two independent MCMC chains consisting of 30,000,000 steps were run and sampled every 3,000 steps. Samples were combined after removing 10% of samples as burn‐in. To account for phylogenetic uncertainty, pairwise evolutionary distances were calculated for each of 100 trees drawn from these posterior samples of trees using the function cophenetic.phylo from the *ape* R package v5.4 (Paradis & Schliep, 2019). Mean pairwise evolutionary distances were calculated averaging across these and used for further analysis.

## 3 DATA ANALYSIS

### 3.1 Ordination and cluster analysis

The ordination of H5N8 Italian outbreaks was achieved using non‐metric multidimensional scaling (NMDS). This is a distance‐based method, which attempts to represent as closely as possible the pairwise dissimilarity between objects, expressed by ranks. Thus, rather than object A being *x* units distant from object B and *x + 1* units distant from object C, object B is the ‘first’ most distant from object A while object C is the ‘second’ most distant. In the final ordination graph, the closer the points are (in the present study the points are the outbreaks), the more similar.Rank‐based methods are generally more robust for data that do not have an identifiable distribution. Any dissimilarity coefficient or distance measure may be used to build the distance matrix used as input for NMDS (Borcard, Gillet, & Legendre, 2011). The goodness‐of‐fit criterion, which measures the degree of distortion of the ordination with respect to the original input data, is called *stress*. Conventionally, *stress* values < 5% indicate a good fit for the data, whereas stress values > 20% give unreliable results. NMDS is also known as unconstrained ordination or indirect gradient analysis, as any information about the environment is not directly included in the analysis, but can be used afterwards as an interpretative tool of the final configuration graph.

We summarized the dissimilarities between the H5N8 outbreaks by computing an Euclidean distance matrix with respect to three main features: the geographical location (space), date of onset of symptoms (time) and HA gene sequence (genetic) of virus isolates. Geographical coordinates serve as input for the space feature and pairwise evolutionary distances, calculated as the sum of branches lengths from the reconstructed time‐measured phylogenies (see *Phylogenetic analysis*), accounted for both temporal and genetic differences between outbreaks. Ordination and corresponding plot were obtained using the *metaMDS* function in the R *vegan* package v2.5‐4 (Oksanen et al., 2019).

An agglomerative hierarchical cluster analysis (function *hclust* in R)—with an unweighted group‐averaging linkage method—was applied to the same distance matrix used as input in NMDS. A cophenetic correlation coefficient has been calculated as a measure of how accurately a dendrogram reflects the pairwise distances between the original unmodelled data points. If this value is high, it derives that the dendrogram is an appropriate summary of the data. Optimal number of clusters was chosen based on the graph of silhouette width (function *silhouette* R package *cluster*), coupled with the ecological interpretability of the groups, as suggested by Borcard and colleagues (Borcard et al., 2011). Clustering and ordination analyses were compared to check the adequacy and mutual consistency of both representations. Once identified, the clusters were used as a categorical outcome variable in the last step of the data analysis, consisting of a multivariable regression. The ordination and cluster analysis were repeated with the phylogenies calculated using an alignment of the 83 domestic cases sequences only, to check whether the inclusion of wild birds cases would affect the results.

We used box and whisker plots to illustrate the variability of each environmental variable among the clusters and tested the differences using Kruskal–Wallis test (KW) and post hoc pairwise comparisons based on Dunn test (Dt) with Bonferroni's adjustment of the p‐value.

### 3.2 Multivariable regression: multi‐model selection and inference

When faced with a biological question, such as to determine which covariates are driving an ecosystem, one of the most difficult aspect of the data analysis is probably how to deal with collinearity, which implies that two or more regressors are conveying the same information (Zuur, Ieno, & Elphick, 2010). Before running the multivariable regression, we addressed this issue by sequentially dropping the covariate with the highest variance inflation factor (VIF), recalculate the VIFs and repeat this process until all VIFs were smaller than 5. When the variable with the highest VIF had an alternative measure of the same predictor (e.g. EVI and NDVI), then the more biologically plausible variable from this pair, or the one which gives a better measure of the relative quality of the regression model for a given set of data (i.e. lower Akaike information criterion, AIC), was chosen to be retained. Then, a multinomial logistic regression model was generated with the package *nnet* v7.3‐12 (Venables & Ripley, 2002) with the putative predictor non collinear variables as covariates, and the clusters of HPAI H5N8 outbreaks identified with the cluster analysis as the outcome variable. Cluster one was used as reference category. Model selection was based on Information Theoretic approach using the corrected Akaike Information Criterion (IT‐AICc) (Burnham & Anderson, 2004). This inferential approach, known as multi‐model selection, is increasingly recognized as an alternative to the use of null hypothesis testing (Burnham & Anderson, 2004; Grueber, Nakagawa, Laws, & Jamieson, 2011). It allows exploring a comprehensive set of potential models obtained as a result of multiple combinations of the explanatory variables. So, instead of considering a unique final ‘best’ model, as it is the case in classical forward, backward, or stepwise model selection procedures, with multi‐model selection it is possible to identify a set of ‘top models’ that can be ranked and weighted according to information criteria. In case of uncertainty in model selection, model averaging within this set of top models provides quantitative measures of each variable's relative importance and allows obtaining robust parameter estimates while addressing the uncertainty associated with them (Burnham & Anderson, 2004). This methodology can be particularly appropriate in the study of complex ecological systems, where multiple interactions take place, and the interest is in finding strong and consistent predictors of a particular outcome.

Once the global model (i.e. the multinomial logistic regression model) has been supplied, a set of potential models, obtained as a result of all possible combinations of the explanatory variables, was generated. For each model, the Akaike weight (wAICc), a measure of the probability that a model is the most likely, was computed and used to make comparison with the other models. Within the set of all the potential models, only those whose total cumulative Akaike weights (wAICc) was at least equal to 0.95 were selected for model averaging. By doing this, it can be inferred that the selected models include the AIC‐best model with a probability of 0.95. In the model with averaged coefficients, variable importance is calculated as the cumulative wAICc of the models in which the variable was included as predictor. This value can be interpreted as the probability that the variable in included in the best model. Multi‐model selection of the multinomial model was performed using the *dredge* function from the package *MuMIn* v1.42.1 (Barton, 2018) in R statistical software v3.5.3 (R Core Team, 2019). The package *AICcmodavg* v2.2‐2 (Mazerolle, 2019) was used to estimate model‐averaged parameters, unconditional standard errors and 95% CIs.

To find evidence of fine‐scale spatial variation that was not accounted for in the model, plots of the empirical variogram of the standardized residuals for each of the multinomial models whose total cumulative wAICc was at least equal to 0.95 were examined. This was coupled with the computation of envelopes using data permutation (simulations *n* = 999) under the assumption of no correlation. If the variogram plot falls within the envelopes, the model is presumed to have considerably reduced spatial autocorrelation. The empirical variogram and the variogram envelopes were estimated using the *geoR* package in R.

Lastly, to visually examine the gradient of effect of the variables included in the best‐ranked regression models across the ordination graph, smooth surface thinplate splines were fitted using the *ordisurf* function in R package *vegan*. This procedure uses generalized additive models (GAMs) to overlay a smoothed response surface to the ordination space, which allows a more detailed visual interpretation and reveals more complex patterns than just a linear relationship.

## 4 RESULTS

### 4.1 Outbreaks ordination and cluster assignment

**Figure 1:**
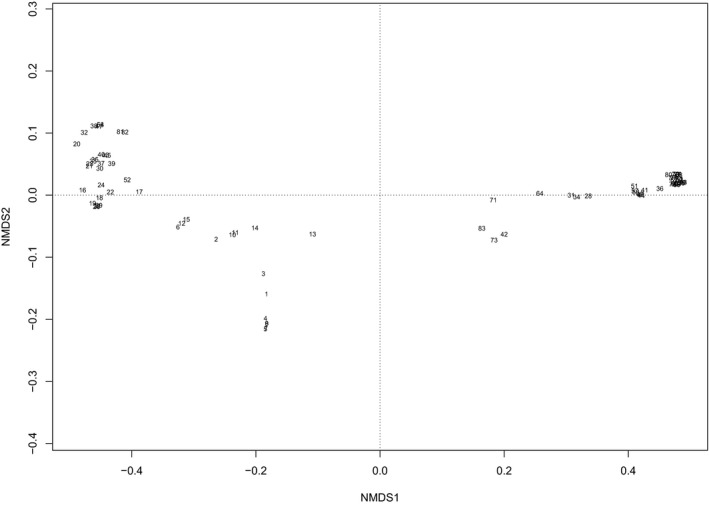
Non‐metric multidimensional scaling (NMDS) plot (Euclidean distance‐based) of H5N8 HPAI outbreaks in Italy in 2017. NMDS1, non‐metric multidimensional scaling dimension one; NMDS2, non‐metric multidimensional scaling dimension two

The NMDS analysis produced a two‐dimensional solution with a final stress value of 1.29%, indicating that the ordination represented well the spatiotemporal and genetic differences among the 83 H5N8 HPAI outbreaks. The ordination graph, given in Figure 1, shows a clear pattern of the outbreaks included in the first and second epidemic waves and, within the latter, a clear separation of two groups. The first wave outbreaks are located in the lower left quadrant of the plot forming a Y shape. The second wave outbreaks, which encompasses all cases where Italy A and Italy B viruses were isolated, laying in the left (Figure S1 & S2) and in the right quadrants (Figure S2), respectively. They comprise as well the four clusters of secondary cases as detailed in Table 1: Man: Mantova; Vic: Vicenza; Ber: Bergamo; Bre: Brescia, and highlighted in Figures S2 and S3. As the results did not change significantly with the use of phylogenies calculated using sequences of domestic cases only, no further results are shown for that data analysis.

**Figure 2:**
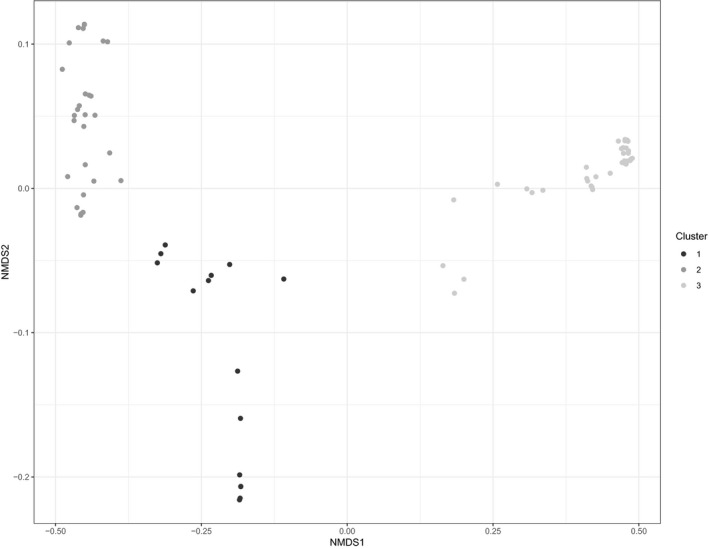
NMDS plot with data points (outbreaks) colur‐coded on a grey scale. Cluster 1 = black, includes India, Croatia and Poland‐like viruses; Cluster 2 = dark grey, includes Italy A virus group; Cluster 3 = light grey, includes Italy B virus group

The outbreaks were assigned to three clusters based on the silhouette width and ecological interpretability. Cluster 1 included outbreaks confirmed between January and April (first epidemic wave), at the time when the India/Poland/Croatia‐like viruses were circulating in holdings located on the skirts of the DPPA. Cluster 2 and 3 included outbreaks confirmed from May to December (second epidemic wave), in holdings where Italy A and Italy B virus groups were isolated, respectively. Figure S4 shows the final dendrogram with boxes around the three clusters. A cophenetic correlation coefficient of 0,95 indicates that the cluster analysis well represent the original distance matrix. Clusters 1, 2 and 3 are arranged by colour code and superimposed on the ordination plot in Figure 2, showing a consistent representation of the spatial, graphical output of ordination and the hierarchical cluster assignment.

In Figure S5, box plots of the variables distribution that differ between clusters are displayed. Examining the differences: (i) distance to the nearest wetlands (KW, *p* = .031), was significantly higher in cluster 3 than cluster 1 (Dt, adjusted *p* = .025); (ii) NDVI vegetation index (KW, *p* < .001) was significantly higher in cluster 2 and 3 compared to cluster 1 (Dt, adjusted *p* < .001 and *p* = .012, respectively); (iii) poultry density and poultry farm density within 3 km (KW, *p* < .001) was significantly higher in cluster 2 and 3 compared to cluster 1 (Dt, adjusted *p* < .001); (iv) poultry population density within 10 km (KW, *p* < .001) was significantly higher in cluster 2 and 3 compared to cluster 1 (Dt, adjusted *p* > .01 and *p* < .001); (v) poultry farm density within 10 (KW, *p* < .001) was significantly higher in cluster 3 and 2 compared to cluster 1 (Dt, adjusted *p* < .001); (vi) cumulative rainfall (KW, *p* < .01) was significantly higher in cluster 2 than in cluster 1 (Dt, adjusted *p* < .01) and in cluster 3 compared to cluster 2 (Dt, adjusted *p* = .02); (vii) the number of rainy days (KW, *p* < .001) was higher in cluster 2 than in cluster 1 (Dt, adjusted *p* < .01); and (viii) elevation (KW, *p* < .001) was higher in cluster 3 compared to cluster 1 and 2 (Dt, adjusted *p* < .01).

Within 1 km around each outbreak locations, the dominant land cover class is agricultural areas, followed by artificial surfaces (well‐developed roads and railways) and a relatively higher percentages of forest and semi‐natural areas in the third cluster, with the latter feature resembling the higher percentage of outbreaks involving the rural sector, located in more hilly areas. None of the land cover classes resulted significantly different between clusters.

### 4.2 Regression analysis

Multi‐collinearity was high (VIF > 5) among variables representing alternative measurements of animal host density, as well as within the group of eco‐climatic variables, as well as between land cover variables and elevation. The two vegetation indexes were highly collinear. We decided to keep the NDVI because it is a better predictor of the plants growth traits compared to EVI. Amount of precipitation and number of precipitation days were as well highly collinear; the latter was kept because it was a better predictor than the previous. At last, elevation was found highly collinear with two land cover classes: with agricultural areas, showing a negative correlation coefficient (−0.629), and with forest and semi‐natural areas, showing a positive correlation coefficient (0.923). However, elevation was preferred because it was repeatedly found associated with a lower risk of AI occurrence in the literature (Gilbert & Pfeiffer, 2012; Mannelli et al., 2006; Si et al., 2010) and because it better predict the outcome variable in the regression analysis compared to the combination of agricultural areas and forest and semi‐natural areas land cover classes. The following variables were omitted from further analysis in order from first to last: EVI, poultry farm density within 10 km, forest and semi‐natural areas, poultry density within 10 km, human density and poultry farm density within 3 km, amount of precipitation, water bodies, rice fields, wetlands and agricultural areas.

The multinomial regression model included 6 predictor variables: poultry population density within 3 km, artificial surfaces, distance to the nearest wetlands, elevation, NDVI and the number of precipitation days.

Sixty‐four models were built considering all the possible combination of variables. The highest ranked model had a wAICc of 0.43, and 5 models were required to reach a cumulative wAICc of 0.95 (Table 3). Within the multi‐model inference framework, it was possible to quantify the relative variable importance, by estimating the probability that a given explanatory variable is included in the best IT model (Table 4). The model with averaged parameters showed that only few factors suffice to discriminate the outbreaks observed in the H5N8 epidemic of 2017: host density, vegetation index (NDVI) and elevation, which were always included in the top 5 models (out of a set of 64) that accounted for a cumulative wAICc ≤ 0.95. The climatic variable ‘number of rainy days’ was included only in 3 out of 5 models. In contrast, the other factors considered (the percentage of land covered by artificial surfaces and the distance to the nearest wetlands) were of relatively low importance (Table 4), as can be inferred from their lower probability of being included in the best AICc model.

**Table 3:** Ninety‐five per cent confidence set of best‐ranked regression models (the 5 models whose cumulative Akaike weight, wAICc, is ≤0.95)

Candidate models	AICc^†^, ^1^	∆AICc^*^, ^2^	wAICc^3^
poultry density 3 km, elevation, NDVI, no. rainy days	129.58	0.00	0.43
poultry density 3 km, elevation, NDVI	129.82	0.24	0.38
poultry density 3 km, distance to wetlands, elevation, NDVI, no. rainy days	133.16	3.58	0.07
poultry density 3 km, artificial, elevation, NDVI, no. rainy days	133.39	3.80	0.06
poultry density 3 km, artificial, elevation, NDVI	133.59	4.00	0.06

^1^ AICc: corrected Akaike Information Criterion.

^2^ ∆AICc: change in AICc.

^3^ wAICc: AICc weight.

**Table 4:** Multinomial logistic regression with averaged parameters estimates, 95% CI, standard error (*SE*) and variable importance

Outcome	Coefficient	Estimate	95%CI	*SE*	Importance
Cluster 2^1^	(intercept)	2.13	−0.55; 4.82	1.37	
	Poultry population density 3 km	4.02	0.97; 7.07	1.56	1
	Elevation	−3.36	−7.17; 0.44	1.94	1
	Distance to nearest wetlands	0.95	−0.51; 2.42	1.28	0.07
	Artificial surfaces	0.44	−0.37; 1.25	0.41	0.12
	NDVI	2.31	0.89; 3.72	0.72	1
	No. of rainy days	1.28	0.09; 2.47	0.61	0.56
Cluster 3^1^	(intercept)	3.63	1.12; 6.14	1.28	
	Poultry population density 3km	3.73	0.71; 6.75	1.54	1
	Elevation	1.34	−0.61; 3.29	0.99	1
	Distance to nearest wetlands	0.60	0.61; 1.82	0.97	0.07
	Artificial surfaces	0.17	−0.60; 0.94	0.39	0.12
	NDVI	1.12	−0.15; 2.38	0.65	1
	No. of rainy days	1.00	−0.12; 2.12	0.57	0.56

^1^ Cluster 1 is the reference category.

Pattern of community similarity, for both Italy A (Cluster 2) and Italy B (Cluster 3), was strongly and positively influenced by the host density, precipitation and NDVI, compared to outbreaks of the first wave (Cluster 1), the reference category. Diversity between clusters is reflected as well in the altitude. An increase in altitude can be found associated to Cluster 3 compared to Cluster 1, but conversely for Cluster 2 compared to Cluster1. Artificial surfaces and distance to the nearest wetlands contribute to explain the pattern of AI outbreaks, but with a lower relative importance. A higher percentage of artificial surfaces and increasing distance to the nearest wetlands are associated to the probability of belonging to Cluster 2 and 3 than to Cluster 1. The same relationship holds for the distance to the nearest wetlands, for which a higher probability of belonging to Cluster 2 and 3 than to Cluster 1 is associated to a greater distance to the nearest wetlands.

The variograms of the residuals for the 5 models whose cumulative wAICc was 0.95 were all contained by their envelopes, which suggests that no spatial autocorrelation greater than that which might be expected by chance remained after adjusting for the covariates. Therefore, models that account for spatial dependency were not explored in this study.

The GAM approach revealed a complex pattern in the relationship between the outbreaks and most of the environmental variables, with the exception of precipitation days and percentage of artificial areas, for which the algorithm fitted a linear trend. Figure S6 shows how those variables (response surfaces) changes across the space of the NMDS graph, where the ordinated outbreaks are plotted. A decreasing gradients of the variables values can be observed when moving from the left and from the right side of the graph, where Cluster 2 (Italy A) and Cluster 3 (Italy B) lays, respectively, towards the centre, where lays Cluster 1 (India/Poland/Croatia‐like). Explained deviance was ~40% for the elevation, NDVI and poultry population density, suggesting that the models have a relatively high explanatory power and predictability. These variables were also the ones with the highest importance according to the multi‐model selection inference. Lower values of explained deviance were obtained for distance to the nearest wetlands (22.6%), number of precipitation days (11.2%) and artificial surfaces (7.08%). The fitted response surfaces were significant for all the models.

## 5 DISCUSSION

Framing avian influenza outbreaks features against variations in eco‐environmental variables is a first and necessary step towards identifying whether different contexts might support distinct epidemiological processes underlying viral introduction and spread in a naïve poultry population. In the present study, the integration of different dimensions of the ecological system (space, time and HA genetic variability), within which H5N8 HPAI occurred in the domestic poultry in Italy in 2017, have been considered and analysed with a combination of multivariate statistical and regression techniques adopted from the field of environmental and ecological sciences. The composition of the outbreaks was hypothesized to vary along gradients of eco‐environmental factors as the underlying introduction and spreading mechanisms of AI virus changed.

The ordination and cluster analysis consistently discriminated 3 groups within the outbreaks community, which resulted associated to opposite environmental gradients in the multinomial regression analysis. In the ordination diagram (Figure 1), outbreaks belonging to the first wave, with the exception of the last one recorded in May (outbreak ID: 16 in Table 1) that clusters with the Italy A group, are more distant from each other and hence more dissimilar compared to the outbreaks of the second wave. In fact, those two groups differed in a number of ways (Mulatti et al., 2018). Firstly, in terms of time frame (date of onset of symptoms), outbreaks of the first wave did not follow the typical avian influenza pattern of occurrence, with few cases reported per week. Secondly, they were located in the fringes of the DPPA (not in close proximity to each other). Thirdly, genetically they include viruses related to three different introductions occurred in Italy at the beginning of the Italian H5N8 HPAI epidemic: H5N8‐A/wild duck/Poland/82 A/2016‐like, H5N8‐A/painted stork/India/10CA03/2016‐like, H5N8‐A/mute swan/Croatia/70/2016‐like (Fusaro et al., 2017). In the NMDS space, a high degree of distinctiveness between Italy A and Italy B groups of outbreaks is well represented (they are located in two different and opposite quadrants) along with a clear separation from the outbreaks of the first wave (located in between the Italy A and Italy B groups). Indeed, genetic analyses (Fusaro et al., 2017) and further integrated phylogenetic and phylodynamic analyses (Harvey et al., 2019), suggested that Italy A and Italy B groups likely originated in Italy and emerged when the H5N8 virus was already circulating in this country, prior to the first case of the second wave. In addition, the outbreaks ascribed to Italy A and Italy B differed in the geographical locations where they occurred, which never overlapped during the epidemic (Mulatti et al., 2018). Italy A was isolated in outbreaks affecting the eastern part of the DPPA, while Italy B circulated mainly in the western part of that area, with sparse incursions in the southern regions involved in the epidemic in 2017. Contrarily, the similarity shown in the ordination graph within the Italy A and Italy B groups is very high, and it is possible to pinpoint multiple areas where points overlapped. Those correspond to the 4 different clusters inferable as such by Mulatti and colleagues (Mulatti et al., 2018) for the high number of connections (proximity between cases, genetic similarity and/or trade of living poultry).

Using multi‐model selection, a combination of host density and eco‐climatic variables, rather than land cover predictors, appeared to best model the probability of belonging to one of the three clusters of outbreaks identified in this study, with a ruling effect of poultry population density within a 3km radius, NDVI and elevation, followed by precipitation, presence of artificial surfaces and distance to the nearest wetlands. In addition, the response surfaces produced for each factor using additive models, showed that outbreaks belonging to the first epidemic wave (Cluster 1) are consistently associated to lower values (decreasing gradients) for predictors such as poultry density within 3 km, NDVI, elevation, distance to the nearest wetlands and artificial surfaces percentage (Figures S6). This strongly contrasts with the highest values (increasing gradients) of the same variables that resemble the ordination pattern of Italy A and Italy B type outbreaks in the second wave (Cluster 2 and 3).

Resuming the conceptual framework that tries to relate avian influenza outbreaks pattern to different drivers of the pathogen introduction and spreading routes, it posits that if AI virus is introduced into the domestic poultry via wild birds, the outbreaks pattern could be found related with features that promote wild bird host ecology (i.e. breeding and feeding habits). On the contrary, when the pattern is related to human related activities or some proxies for that, a secondary between holdings transmission hypothesis can be corroborated (Si et al., 2010, 2013). In the present study, by relating epidemic‐wide gradients of the eco‐environmental factors to outbreaks community, findings suggested that first wave outbreaks ecologies are more capable of supporting AI potential incursions from wild birds into the domestic poultry sector. As opposite, ecologies of the second wave outbreaks more likely support a predominant lateral spread mechanism (farm‐to‐farm).

Importantly, environmental correlates of the Italian H5N8 outbreaks dissimilarity pattern match earlier observations made for defining H5N1 HPAI virus persistence and area suitability (Belkhiria, Hijmans, Boyce, Crossley, & Martínez‐López, 2018; Gilbert & Pfeiffer, 2012; Wang et al., 2014; Ward, Maftei, Apostu, & Suru, 2008), while giving further evidence to the more recent study by Dhingra and colleagues (Dhingra et al., 2016). The latter indeed concluded that a combination of host distribution and eco‐environmental variables define a better suitability model for the H5Nx clade 2.3.4.4 AI viruses, compared to the model for the H5N1 HPAI, which includes host distribution variables only.

Notwithstanding, it is possible to pinpoint factors—elevation, distance to nearest wetlands and number of rainy days—with respect to whose gradients, Italy A and Italy B can be discriminated as a sort of sub epidemics. Higher altitudes, have always been found negatively associated to the risk of outbreaks occurrence (Gilbert & Pfeiffer, 2012; Mannelli et al., 2006; Si et al., 2010), possibly because of increased geographical isolation, which implies local environmental conditions less suitable for virus spread. In the present study, elevation resulted positively related to outbreaks of Italy B type. While there is no apparent explanation, this condition may simply reflect the higher percentages of outbreaks belonging to the rural poultry sector where this genomic groups was isolated and that were located at a relative higher altitude than the median of the same group. Besides, it should not be forgotten the fact that elevation showed a high and positive correlation with land cover class ‘forest and semi‐natural areas’ and a negative one with ‘agricultural areas’, which simply convey the same information as the elevation variable does. Vegetation characterized by larger plants such as bushes and forest trees (as in the forest and semi‐natural areas) are not suitable for waterfowl feeding behaviour, thus strengthen the hypothesis that those outbreaks were more likely related to a secondary farm‐to‐farm transmission.

Variables indicative of the presence of water, although always associated with HPAI outbreaks, have usually lower statistical significance levels compared to host or human densities factors (Si et al., 2010, 2013; Wang et al., 2014). However, the ‘distance to nearest wetlands’ variable shows the direction of maximum increase towards the Italy B genomic group outbreaks and the model surfaces overlay the Bergamo cluster (Figures S3 and S6). This cluster comprised all rural farms showing as well higher values of altitude variable.

A positive gradient of the number of rainy days in the 15 days preceding the onset of symptoms is found associated to outbreaks of Italy A type. Rainfall patterns may importantly determine breeding opportunities and are therefore linked to wild bird numbers (Si et al., 2010). This subsequently can influence the age structure of that community, which may well affect AI dynamics. However, we cannot rule out that rainfall may also have a direct effect on AI dynamics, as AI virus is generally highly persistent in water (Stallknecht, Shane, Kearney, & Zwank, 1990). These findings contrast with the hypothesis that relates cluster of Italy A type outbreaks to variables that are proxies for a secondary farm‐to‐farm transmission. However, phylogenetic and temporal distances have already suggested the persistence of the Italy A genetic group in wild birds, with a higher number of primary incursions into domestic poultry compared to what was reconstructed for Italy B virus group (Harvey et al., 2019). The results of the approach used in the present study provide further evidence that the role of wild birds during the Italian epidemic was significantly greater than apparent.

Surprisingly, of all the land cover variables considered, with the exception of artificial surfaces, showing a relatively low importance in model prediction, none was retained in the best subset of models. Most probably, this result reflects a limitation of our choice in defining the buffer area for data extrapolation. One kilometre buffer could have been a too small area to capture relevant features that can be related to a source of infection due to proximity. This translated as well in strong discontinuities along the data set of such variables. In cases like this, methods which set a threshold (e.g. regression or classification trees) would have been better at describing the relationship between these predictors and the clusters than linear models does (Ouellette, Legendre, & Borcard, 2012).

This study offered the opportunity to explore the peculiarities of the Italian H5N8 HPAI epidemic using a disease ecology perspective. The results corroborate the hypothesis that, in Italy, the H5N8 HPAI epidemic occurred within the domestic poultry population in 2017, owed their distinctive marks to different epidemiological processes that shaped the virus introduction and further spread (Harvey et al., 2019; Mulatti et al., 2018). However, to date the processes linked to the emergence in the winter and recrudescence in summer of 2017 of the H5N8 HPAI in Italy remain difficult to unravel, not least for the lack of recent and updated data on consistency and distribution of waterbirds populations wintering in this country, as well as data related to the residential waterfowl populations. This makes it difficult to establish whether important changes, if any, have occurred with regard to the populations’ size and species habits, and whether these, along with local environmental changes, exerted an impact on the spread of the disease. Suggestive evidence of the potential role of residential wild birds in maintaining and spreading influenza viruses can be found in the annual waterbirds census carried out in the lagoons of Venice, undertaken every year as part of the IWC (International Waterbirds Census), which reported a seven fold increase in the migratory waterfowl population from 1993 to 2018 (personal communication *Scarton F.; SELC Soc. Coop* www.selc.it).

One important advantage in our study is that outbreaks community patterns were derived from empirical data, rather than spatially extrapolated on the basis on environmental relationships or assumed ecological niches. Besides, phylogenetic data were integrated in order to consider virus evolution during the epidemic as a pivotal dimension of the epidemic ecological system. The present study has considerable potential, and its insights could be applied to initiate diverse modelling approaches in order to more accurately predict disease occurrence at different space and time scales associated to ecological variation, which are often mediated by complex, large‐scale processes that are not immediately amenable to traditional approaches to causal inference. Two steps should be undertaken in any follow‐up. Firstly, with more phylogeographic studies becoming available, it should be possible to separate true persistence in a country from new introductions, which would enhance our capacity to identify the areas most susceptible to those events. In conjunction with the recent advent of phylogenetic trait‐based approaches, other ecological analysis frameworks may be considered to tackle ecological data assessment, when the relationship between a main explanatory data set and the response is non‐linear (Ouellette et al., 2012; Zuur, Ieno, & Smith, 2007), such as niche modelling, multivariate regression trees or classification trees and their extensions (i.e. boosted regression trees). Secondly, we could have more finely estimated risk indices at farm level. For example, biosecurity practices, trade activities and inbound and outbound movements data often hide considerable within country variation. It can be anticipated that they may prove useful in refining several site‐specific conditions where AI control efforts may be targeted. Similarly, it would also be important to consider how biological and climatic processes that help explaining outbreaks pattern in domestic poultry, interact with the dynamics of AI infection in the wild bird population, and the practical applications of this for surveillance programmes.

Importantly, eco‐environmental conditions are dynamic and so are the interactions between hosts and the environment where avian influenza (AI) viruses circulate. Expanding temporal and spatial coverage to historical data of Italian LPAI and HPAI epidemics or to the European H5N8 HPAI most recent epidemic data may help identify trajectories over which some countries, or peculiar poultry production systems develop greater risk for AI incursions and persistence, and to fully describe the environmental gradients in which influenza viruses may evolve.

## CONFLICT OF INTEREST

The authors declare no competing interests.

## ETHICAL APPROVAL

The study did not include any experimentation on animals or humans. Samples were taken from dead birds on infected farms, as part of measures provided by the European Council Directive on Avian Influenza (Council Directive 2005/94/EC).

## Supporting information

Fig S1‐S6

## Data Availability

The data supporting the findings of this study are available from the corresponding author upon reasonable request.
