# Supplementary material for: Different environmental gradients associated to the spatiotemporal and genetic pattern of the H5N8 highly pathogenic avian influenza outbreaks in poultry in Italy
Source: Transbound Emerg Dis. 2020 Jul 2;68(1):152–67. doi: 10.1111/tbed.13661 (PMC8048857; doi:10.1111/tbed.13661)
Supplement: Supplementary file 1 — Fig S1‐S6 [file TBED-68-152-s001.pdf]

Supplementary Material

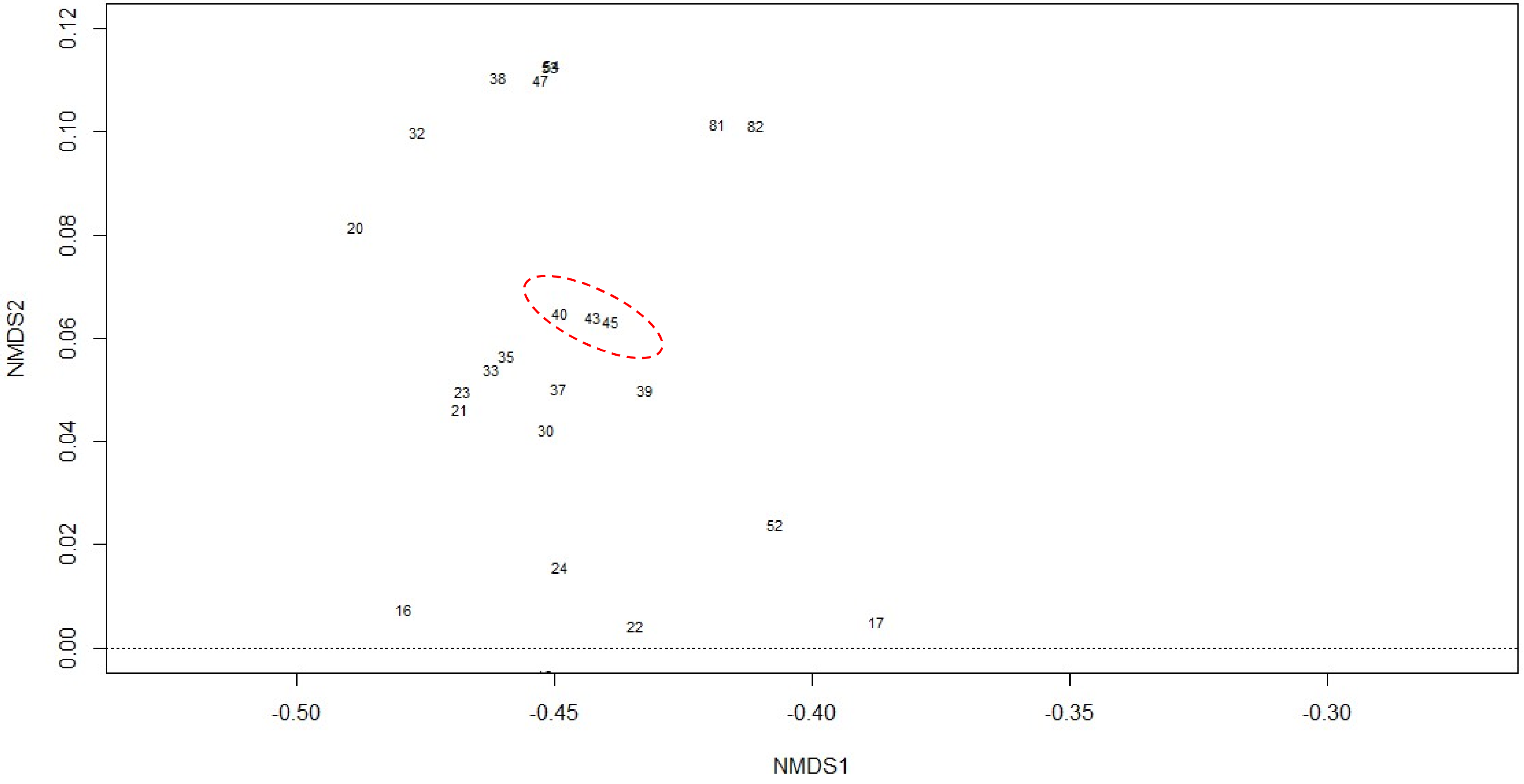

Figure S1. Zoom on the Italy A group in the upper left quadrant of the NMDS plot (Figure 1 in the manuscript), comprising the Vicenza cluster of secondary cases (red dashed ellipse).

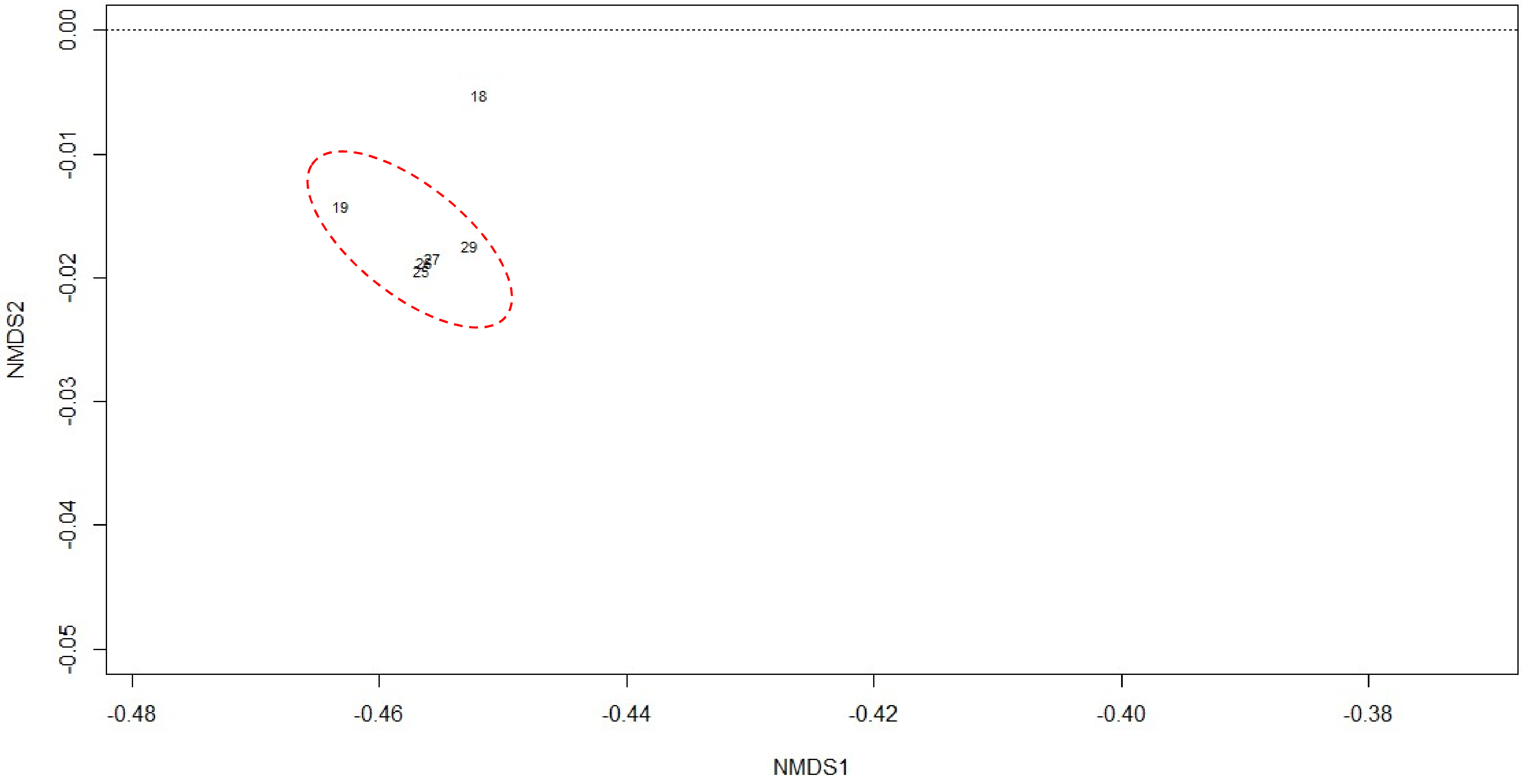

Figure S2. Zoom on the Italy A group in the lower left quadrant of the NMDS plot (Figure 1 in the manuscript), comprising the Mantova cluster of secondary cases (red dashed ellipse).

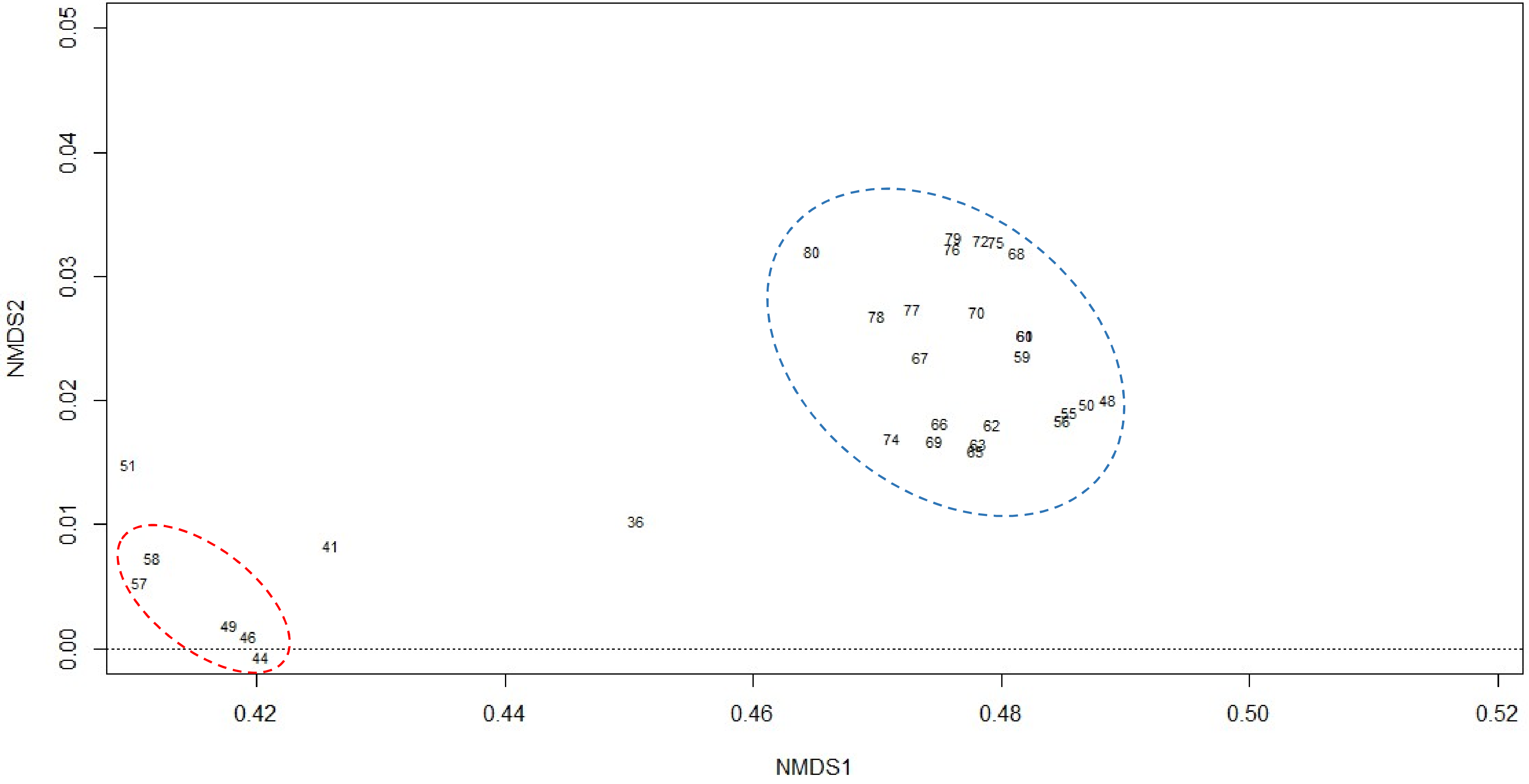

Figure S3. Zoom on the Italy B group in the upper right quadrant of the NMDS plot (Figure 1 in the manuscript), comprising the Bergamo (red dashed ellipse) and the Brescia (blu dashed ellipse) clusters of secondary cases

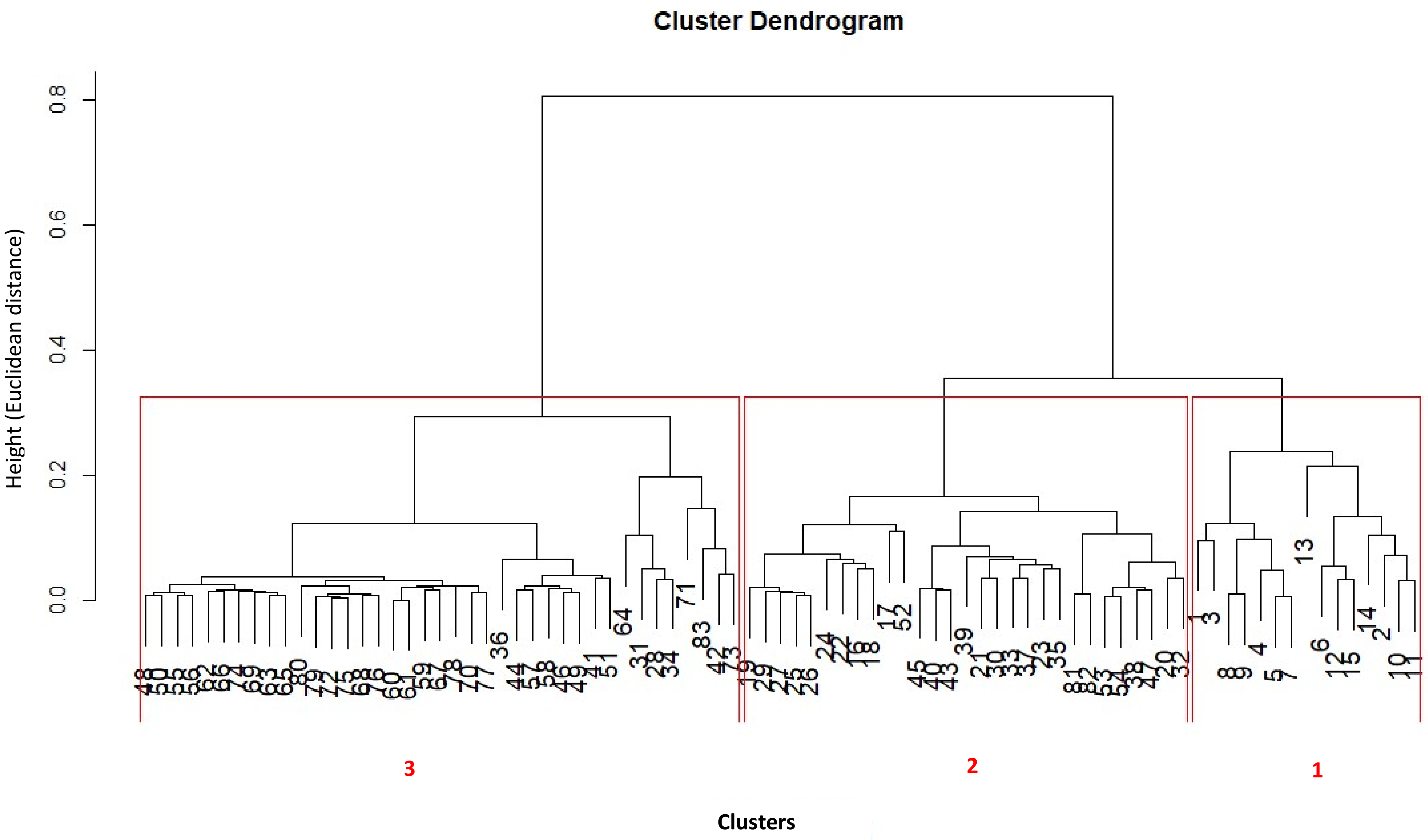

Figure S4. Results of hierarchical cluster analysis presented as a dendrogram and with rectangles identifying optimal number of clusters. Average linkage agglomerative clustering of the matrix of Euclidean distances among sites (outbreaks data) was used.

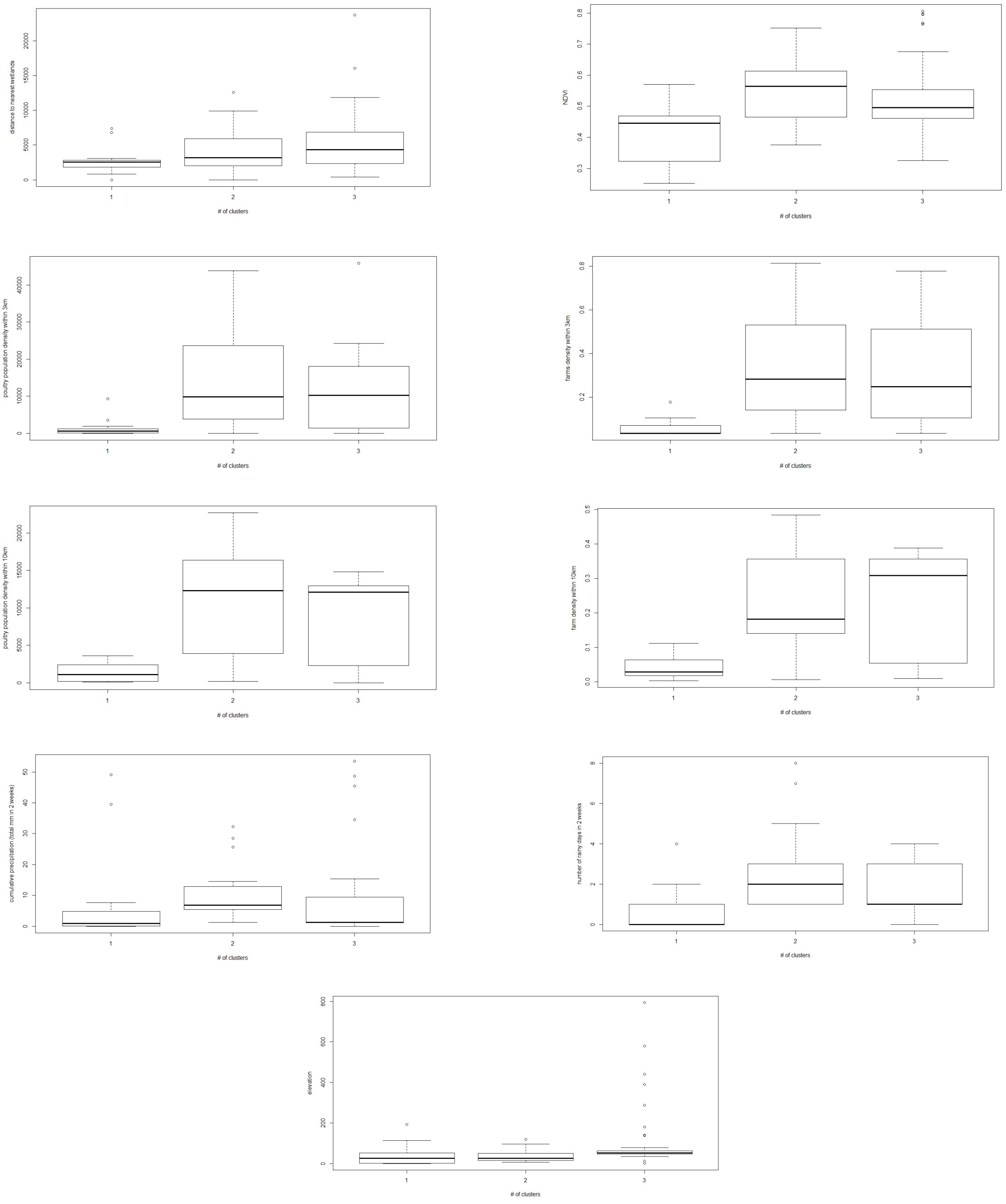

Figure S5. Box-plot diagrams showing how the eco-environmental factors vary between clusters

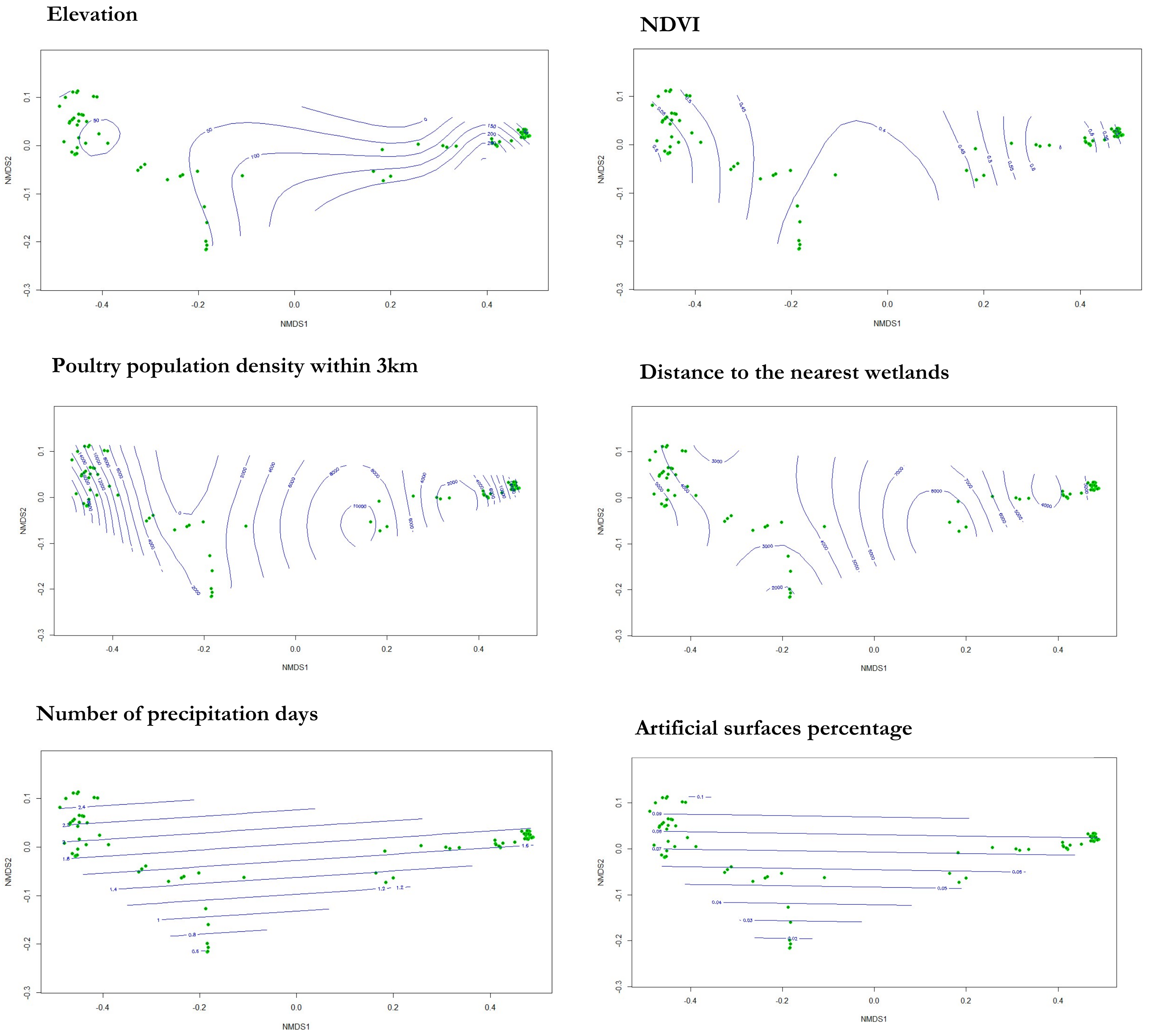

Figure S6 Ordinations of the H5N8 HPAI outbreaks data with response surfaces for the environmental variables selected in the best ranked multinomial regression**.** Numbers on the splines represent value of the specific environmental variable.
